# The two-hit hypothesis for neuroinflammation: role of exogenous ATP in modulating inflammation in the brain

**DOI:** 10.3389/fncel.2014.00260

**Published:** 2014-09-01

**Authors:** Bernd L. Fiebich, Shamima Akter, Ravi Shankar Akundi

**Affiliations:** ^1^Department of Psychiatry and Psychotherapy, Neurochemistry Research Laboratory, University of Freiburg Medical SchoolFreiburg, Germany; ^2^Neuroinflammation Research Laboratory, Faculty of Life Sciences and Biotechnology, South Asian UniversityNew Delhi, Delhi, India

**Keywords:** ATP, microglia, neuroinflammation, NSAIDs, P2 receptors, PBAIDs, prostaglandin E2

## Abstract

Brain inflammation is a common occurrence following responses to varied insults such as bacterial infections, stroke, traumatic brain injury and neurodegenerative disorders. A common mediator for these varied inflammatory responses is prostaglandin E_2_ (PGE_2_), produced by the enzymatic activity of cyclooxygenases (COX) 1 and 2. Previous attempts to reduce neuronal inflammation through COX inhibition, by use of nonsteroidal anti-inflammatory drugs (NSAIDs), have met with limited success. We are proposing the two-hit model for neuronal injury—an initial localized inflammation mediated by PGE_2_ (first hit) and the simultaneous release of adenosine triphosphate (ATP) by injured cells (second hit), which significantly enhances the inflammatory response through increased synthesis of PGE_2_. Several evidences on the role of exogenous ATP in inflammation have been reported, including contrary instances where extracellular ATP reduces inflammatory events. In this review, we will examine the current literature on the role of P2 receptors, to which ATP binds, in modulating inflammatory reactions during neurodegeneration. Targeting the P2 receptors, therefore, provides a therapeutic alternative to reduce inflammation in the brain. P2 receptor-based anti-inflammatory drugs (PBAIDs) will retain the activities of essential COX enzymes, yet will significantly reduce neuroinflammation by decreasing the enhanced production of PGE_2_ by extracellular ATP.

## Inflammation within the brain

Various environmental factors can lead to inflammation within the brain. These range from bacterial infections that cause acute inflammation to neurodegenerative disorders that mediate chronic inflammation. The inflammation may be restricted to a local region in focal ischemia or occur in a wider zone during traumatic brain injury. Inflammation could also result from an autoimmune response such as multiple sclerosis or in response to toxins and nerve agents (for general reviews, see Lucas et al., 2006; Aguzzi et al., 2013). Recent reports implicate inflammation contributing to the pathology of psychiatric disorders such as stress, depression and schizophrenia (Najjar et al., 2013), in metabolic syndromes such as obesity and type 2 diabetes (Purkayastha and Cai, 2013), and even as a response to increased neuronal activity (Xanthos and Sandkuhler, 2014). Irrespective of the type of inflammation, the molecular mediators are oftentimes the same –prostaglandin E_2_ (PGE_2_) or cytokines such as interleukin-1β(IL-1β), produced by the activity of resident microglial cells. Despite brain inflammation playing such a major role in various CNS disorders, successful therapeutic strategies to overcome it are still lacking.

PGE_2_ is produced by the action of cyclooxygenases (COX) which mediate the first committing step in its synthesis from arachidonic acid (Akundi et al., 2005). The constitutively active COX-1 isoform is believed to be responsible for the majority of PGE_2_ formed in the body. However, it is the growth factor-, cytokine- or mitogen-inducible COX-2 that emerged as the isoform responsible for the massive release of PGE_2_ during inflammation of all types—systemic, central, acute or chronic. The COX enzymes have been a therapeutic target in a multitude of disorders, ranging from fever and pain to cancer, rheumatoid arthritis, and Alzheimer’s disease (AD; Yedgar et al., 2007). Their importance can be judged from the widespread use of aspirin as an analgesic and antipyretic; and the promise of nonsteroidal anti-inflammatory drugs (NSAIDs) against spreading neurodegeneration in AD (Szekely and Zandi, 2010). However clinical trials failed to not only halt the progression of dementia in AD patients but also showed increased risks of myocardial infarction and stroke (Jüni et al., 2004). An essential lesson learnt from the debacle of NSAIDs was that the two isoforms of COX do not functionally substitute one another but each remains indispensable in certain functions. COX-2 stands on a delicate balance—the neuronal isoform plays an important role in synaptic plasticity, memory consolidation and cortical development while the microglial isoform mediates neuroinflammation. Targeting COX enzymes, therefore, requires a careful consideration of the benefit-to-risk ratio.

## Neuroinflammation: a two-hit model

**Figure 1:**
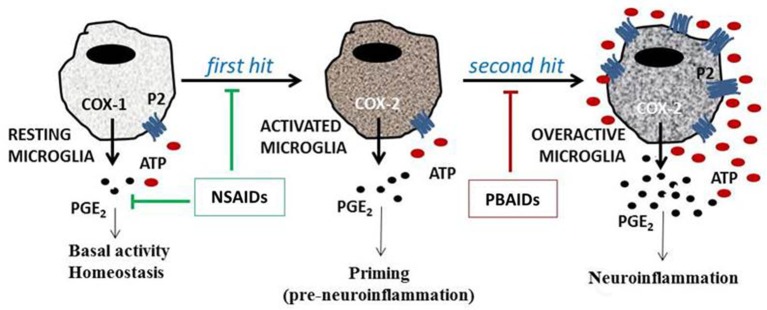
**The two-hit model of neuroinflammation**. The ATP-mediated enhancement of neuroinflammation can be explained through the two-hit model. A variety of insults, such as bacterial LPS, various cytokines, or amyloid peptides, can act as the *first hit*, resulting in microglial activation, COX-2 induction and PGE_2_ release. The *second hit*, following neuronal injury, death or persistent glial cell activation, results in the release of ATP, which acts on both neuronal and glial P2 receptors, leading to enhanced microglial PGE_2_ release. NSAIDs target COX enzymes affecting the housekeeping roles of PGE_2_. ATP potentiates the effects of first hit multi-fold, and thus, would be the most relevant target for therapeutic intervention. By acting on P2 receptors, PBAIDs are believed to reduce PGE_2_ to pre-inflammatory levels without affecting the activity of COX enzymes.

An interesting observation in the past decade and half showed that the inflammatory response of microglia—the release of mature IL-1β from lipopolysaccharide (LPS)-primed cells—could be significantly modulated with the addition of exogenous adenosine triphosphate (ATP; Ferrari et al., 1997). More diversified studies showed that ATP is able to mediate the release of PGE_2_ in IL-1β-treated astrocytes (Xu et al., 2003) or in LPS-activated macrophages (Barberà-Cremades et al., 2012). We found a similar synergistic effect of ATP on LPS-mediated PGE_2_ release in primary rat microglial cells (unpublished observation). These studies conclusively showed that exogenous ATP significantly modulates inflammation. In this review, we are proposing the two-hit model for neuroinflammation. The *first hit* is the injury itself—nerve injury, bacterial infections, hypoxia-ischemia, autoimmune reactions or proteopathies associated with neurodegeneration—leading to the activation of glial cells (Figure 1). The *second hit* is the release of large pools of cytosolic ATP from damaged neurons into the extracellular milieu, in response to direct injury or following glial cell activation. This excess ATP, despite mechanisms regulating their concentration outside the cell, activates a wide variety of purinergic receptors present on cells in the vicinity, thus modulating glial activity and neuronal response to inflammation. Such a model was earlier proposed for the release of mature IL-1β following bacterial infections (Ferrari et al., 2006). Identifying the pro-inflammatory receptors of ATP, and targeting them pharmacologically, will significantly diminish the dramatic release of prostanoids and cytokines to clinically manageable levels; thus, balancing their functional roles in active defence and tissue repair.

## ATP: the common denominator for varied inflammatory insults

Both healthy neurons and glial cells carry ATP, in millimolar concentrations, within presynaptic vesicles and granules, respectively (Abbracchio et al., 2009). Neuronal ATP serves as a neurotransmitter while astrocytic ATP allows distant astrocytes to communicate with each other and modulate neuronal response. However, the release of ATP by neurons or astrocytes is usually very low, in the nanomolar range. Furthermore, the extracellular concentration of ATP is dependent on the regional distribution and local activity of synaptic ectonucleotidases CD28/CD39 which convert ATP to ADP and AMP, CD73 which converts AMP to adenosine, and nucleoside diphosphate kinase whose transphosphorylating activity maintains the exogenous levels of various nucleotides in steady state (Lazarowski et al., 2003). This steady state balance is, however, disrupted during pathological conditions when damaged neurons and chronically activated glial cells release dramatic levels of ATP, uridine triphosphate (UTP) and other intracellular nucleotides.

Not only neuropathological conditions even systemic inflammation leads to an increase in exogenous ATP within the CNS (Gourine et al., 2007). In fact the release of ATP in response to tissue injury is a universal phenomenon also seen in plants at sites of physical wounding (Choi et al., 2014). Efflux of ATP into the extracellular space is a common universal “stress signal”, leading to the evolution of receptors for ATP to recognize this “danger” and initiate a stress response. Mammals evolved purinergic receptors with varying specificities for ligands such as ATP, ADP, UTP, UDP, UDP-sugars or adenosine, and diverse range of intracellular signaling mechanisms downstream to receptor activation. Nucleotides act on P2 receptors, with seven ionotropic P2X receptors gating Na^+^, K^+^, and especially Ca^2+^ ions, and eight G protein-coupled metabotropic P2Y receptors (Abbracchio et al., 2009). Adenosine, on the other hand, acts on adenosine receptors, of which A_1_ and A_3_ adenosine receptors inhibit, while A_2A_ and A_2B_ adenosine receptors stimulate adenylyl cyclase (Fredholm, 2010). Recently ATP receptors have also been identified in plants which are activated in response to tissue wound (Choi et al., 2014). Called DORN1 (Does not Respond to Nucleotides 1), these receptors, much like their mammalian counterparts, show high affinity to ATP and alter Ca^2+^ flow.

## Exogenous ATP has both positive and negative roles in inflammation

The presence of functionally active purinergic receptors on microglia indicates the likelihood of astrocyte-microglia crosstalk (Verderio and Matteoli, 2001). Such a communication enhances microglial surveillance system and their response to inflammation within the CNS. Indeed, neuronal and astrocytic release of ATP during traumatic brain injury causes rapid microglial chemotactic response (Davalos et al., 2005). At the site of injury, exogenous ATP mediates the release of pro-inflammatory cytokines and PGE_2_ (Xu et al., 2003; Ferrari et al., 2006; Xia and Zhu, 2011). The end effect of this synergism is the production of pathological levels of inflammatory cytokines and prostanoids.

While the above reports suggest extracellular ATP as proinflammatory, others have reported to the contrary. In LPS-primed cells, ATP inhibited the release of cytokines from spinal cord microglia (Ogata et al., 2003), nitric oxide (NO) release in BV-2 microglia (Brautigam et al., 2005), and pro-inflammatory markers such as tumor necrosis factor α (TNF-α), interleukin 6 (IL-6) and NO in primary microglia (Boucsein et al., 2003). Since TNF-α and IL-6 also have neuroprotective roles (Suzuki et al., 2004; Noguchi et al., 2013), the above inhibitory action of ATP may actually be detrimental to the organism. Increases in exogenous ATP need not always be hazardous—extracellular levels of adenosine increases 6- to 31-fold within the hippocampus of patients with epilepsy, but acts as a natural anticonvulsant terminating seizure (During and Spencer, 1992). Interestingly, cancer cells evade surveillance by up-regulating a subpopulation of regulatory T cells expressing ectonucleotidases CD39 and CD73 to exploit the immunosuppressive nature of adenosine (Whiteside and Jackson, 2013). Early development of nervous system is dependent on purinergic signals, which in concert with growth factors, regulate the number of proliferating and differentiating neural stem cells (Ulrich et al., 2012).

## P2 receptors determine the eventual effect of extracellular ATP

### P2X_7_ receptors

The specific roles of P2 receptors in neuroinflammation are still being uncovered. The subject is still in its teens, starting with the discovery of ATP enhancing IL-1β release in activated immune cells (Ferrari et al., 1997). LPS-mediated activation of toll-like receptor 4 leads to the formation of the inflammasome complex wherein IL-1β processing occurs (Martinon et al., 2002). However, release of IL-1β requires loading of the inflammasome complex into the secretory lysosome, or the formation of membrane blebs—either mechanism triggered through P2X_7_ receptor-mediated K^+^ efflux (Ferrari et al., 2006; di Virgilio, 2007). As a result, the effect of ATP is dependent on cells primed with LPS, and conversely, LPS does not release IL-1β in the absence of P2X_7_ receptors (Solle et al., 2001). Pannexin 1, a gap junction-related protein, has been shown to be responsible for the release of ATP from dying cells, leading to the activation of the inflammasome and recruitment of phagocytes (Dahl and Keane, 2012). Intraperitoneal injection of LPS results in two-four-fold higher detection of ATP in the mouse peritoneum (Barberà-Cremades et al., 2012). Systemic administration of LPS markedly increases the expression of P2X_7_ receptors in the brain (Choi et al., 2007). LPS- or IL-1β-mediated febrile response is greatly reduced in mice with genetic or pharmacological loss of P2X_7_ receptors (Barberà-Cremades et al., 2012). ATP and the preferential P2X_7_ agonist, 2′(3′)-*O*-(4-benzoylbenzoyl) ATP (BzATP) induce the secretion of cytokines IL-6 and TNF-α in wildtype microglia but not in cells derived from P2X_7_^−/−^ mice (Shieh et al., 2014). These reports underline the importance of P2X_7_ receptors in mediating inflammation, especially in the release of IL-1β (Ferrari et al., 2006). The low affinity of P2X_7_ receptors for extracellular ATP ensures their activation occurs only under pathological conditions where excess ATP is found, further supporting the notion of exogenous ATP as an “alarm” signal.

Immunohistochemical analysis of AD brains reveal significant levels of P2X_7_ receptors colocalized with activated microglia, an observation that was also found in the hippocampus of rats injected with Aβ_1-42_ (McLarnon et al., 2006). Aβ triggers ATP release, membrane permeabilization and IL-1β secretion in wild-type but not in P2X_7_^−/−^ mouse (Sanz et al., 2009). In fact, overexpression of P2X_7_ receptor itself, in the absence of any pro-inflammatory stimuli, can drive the activation and proliferation of microglial cells (Monif et al., 2009). Similarly, exposure to high levels of extracellular ATP can also tilt the signaling mechanism from a P2X7-phosphatidylinositol 3-kinase/Akt-mediated growth pathway to a novel P2X7-AMPK-mammalian target of rapamycin (mTOR)-mediated autophagic pathway, as observed in tumor cells (Bian et al., 2013). Rapamycin reduces neuroinflammation and brain lesions in a mouse model of Leigh syndrome (Johnson et al., 2013). In astrocytes, inhibition of mTOR significantly reduces the stability of inducible nitric oxide synthase (iNOS) mRNA (Lisi et al., 2011). However, in microglia, blocking of mTOR pathway in activated cells leads to enhanced PGE_2_ synthesis (de Oliveira et al., 2012). Activated microglia downregulate microRNA, miRNA-200b, which leads to increased c-Jun N-terminal kinase (JNK) activity leading to increased iNOS expression (Jadhav et al., 2014). These observations suggest that the role of mTOR in neuroinflammation is cell-type specific and depends on both epigenetic factors and the presence of inflammatory stimuli.

Gene expression studies in Aβ-treated microglia derived from human post-mortem brains, in fact, suggest that the expression of pro-inflammatory genes are largely up-regulated at the expense of genes involved in Aβ phagocytosis and removal (Walker et al., 2006). Activated P2X_7_ receptors impair lysosomal function and instead stimulate the release of autolysosomal contents into the extracellular space, possibly leading to the increased secretion of IL-1β or amyloidogenic proteins (Takenouchi et al., 2009). In corollary, silencing of P2X_7_ receptors in Aβ-stimulated cells leads to a decreased release of pro-inflammatory cytokines and a marked increase in the phagocytosis of Aβ_1-42_ peptide (Ni et al., 2013). P2X_7_ receptors, therefore, turn phagocytic (neuroprotective) microglia into inflammatory (neurodegenerative) phenotype.

Expression of P2X_7_ receptors is also up-regulated in Huntington’s disease and amyotrophic lateral sclerosis (ALS; Díaz-Hernández et al., 2009). In microglia isolated from superoxide dismutase SOD1-G93A mutant mouse model of ALS, activation of P2X_7_ receptors enhances oxidative stress (Apolloni et al., 2013). Oxidative stress drives the nitration of 90 kDa heat-shock protein (Hsp90), which mediates cell death through P2X_7_ receptors (Franco et al., 2013). Nitrated Hsp90 is found in the motor neurons of patients with ALS; and as expected, deletion of P2X_7_ receptors prevents the neurotoxic effects of nitrated Hsp90.

Imbalances in energy homeostasis are associated with neurodegenerative disorders (Akundi et al., 2013). Over-activation of poly (ADP-ribose) polymerase 1 (PARP1) contribute towards dopaminergic degeneration in Parkinson’s disease (PD), which is completely absent in PARP1^−/−^ mice (Kim et al., 2013). PARP1 activation leads to depletion of cytosolic NAD^+^. Replenishment of NAD^+^ prevents PARP1-mediated neuronal death (Alano et al., 2010). Exogenous NAD^+^, surprisingly, enters neurons through the dilated P2X_7_ receptor-gated channels, marking a neuroprotective role for the otherwise proinflammatory P2X_7_ receptors.

Among other neuroprotective roles, various *in vitro* models show that activation of P2X_7_ receptors stimulates α-secretase activity leading to the shedding of non-amyloidogenic soluble amyloid precursor protein (APP; Darmellah et al., 2012). On the contrary, inhibition of P2X_7_ receptors in a transgenic mouse for mutant human APP show a significant decrease in the number of amyloid plaques through increased activity of α-secretase (Diaz-Hernandez et al., 2012). Such opposing roles could be best explained with the discovery of a shorter, natural, splice variant of P2X_7_ receptor that exhibits neurotrophic properties (Adinolfi et al., 2010). Though it remains to be investigated, it is probable that the truncated P2X_7_ receptors induce α-secretase activity while the longer isoforms are inhibitory. The factors that mediate the retention or deletion of the C-terminal part of P2X_7_ receptors are not yet known. Such contrasting roles of P2X_7_ receptors have also been identified in other cellular systems such as cancer (Feng et al., 2006). The distribution of short and long isoforms of P2X_7_ within the receptor heterotrimer most likely determines its overall trophic or toxic nature.

### P2X_4_ receptors

An interesting use of neuronal P2 receptors as “flags” for microglial recognition has been reported. In the mutant superoxide dismutase SOD1 mouse model of ALS, degenerating motor neurons typically express P2X_4_ receptors for the recruitment and eventual engulfment by activated microglia (Casanovas et al., 2008). Unlike a typical cell undergoing apoptosis, P2X_4_-positive neurons neither show chromatin condensation nor caspase 3 activity; rather exhibit loss of neuronal NeuN marker and recruitment of microglial cells. It is not just restricted to motor neurons but to other degenerating neurons affected with ALS—serotonergic neurons of raphe nucleus, noradrenergic neurons of locus coeruleus, and Purkinje cells in the cerebellum. In Aβ_1-42_-treated neurons that do undergo caspase 3-mediated apoptosis, increased surface expression of P2X_4_ receptors occurs due to the unique presence of a putative caspase 3 cleavage site within the C terminus region (Varma et al., 2009). Hence, overexpression of P2X_4_ receptors enhances Aβ-induced neuronal death, while receptor inhibition subdues cell death. These reports form the basis for our hypothesis that surface expression of P2X_4_ receptors may serve as markers for degenerating neurons, attracting microglial cells for eventual engulfment.

On the other hand microglial P2X_4_ receptor expression is associated with increased neurophagic activity (Cavaliere et al., 2003). Knocking out P2X_4_ receptors results in poorer microglial activation and loss of PGE_2_-mediated inflammatory pathway (Ulmann et al., 2010). P2X_4_ receptor forms a large conductance pore on the cell surface affecting ionic balance, thus mediating the release of proinflammatory substances. Constitutively, P2X_4_ receptors are trafficked into late endosomes and remain resistant to lysosomal degradation (Robinson and Murrell-Lagnado, 2013). Such a mechanism prevents the “flagging” of healthy neurons or the “activation” of microglia under normal physiology.

### Other P2X receptors

Slow neurodegeneration, following axotomy, shows an upregulation of P2X_1_ and P2X_2_ receptors, synchronous with upregulation of neuronal nitric oxide synthase (nNOS; Viscomi et al., 2004). P2X receptors further mediate translocation of nNOS to the plasma membrane (Ohnishi et al., 2009). In an animal model of PD, dopamine denervation upregulates P2X_1_, P2X_3_, P2X_4_ and P2X_6_ receptors on nigral GABAergic neurons to compensate the loss of dopamine (Amadio et al., 2007). Coincidentally, within the substantia nigra, two out of five groups of GABAergic neurons, but none of the five groups of dopaminergic neurons, express nNOS (González-Hernández and Rodríguez, 2000). Whether P2X and nNOS are upregulated within the same cell during neurodegeneration is not known; however, coordinated activation of purinergic and nitrergic mediators seems a likely event during neuroinflammation.

### P2Y receptors

The metabotropic P2Y receptors play a major role in neuron-glia communication. Neuronal injuries activate astrocytic P2Y_1_ receptors leading to the release of PGE_2_, causing reactive gliosis (Xia and Zhu, 2011), or glutamate, mediating synaptic modulation (Domercq et al., 2006). Blocking of P2Y_1_ receptors therefore reduces glial activity (Davalos et al., 2005) and improves cognitive outcome following traumatic brain injury (Choo et al., 2013). In the AD brain, P2Y_1_ receptors are localized in the neurofibrillary tangles and neuritic plaques (Moore et al., 2000). In contrast, there is a selective loss of P2Y_2_ receptors correlating with worsening neuropathological scores (Lai et al., 2008). This is not surprising since P2Y_2_ receptors stimulate α-secretase activity (Camden et al., 2005). In addition, P2Y_2_ receptors mediate microglial phagocytosis of fibrillar forms of Aβ in a mouse model of AD (Ajit et al., 2014). The soluble Aβ peptides are instead cleared through ATP-dependent P2Y_4_ receptor-mediated pinocytosis (Li et al., 2013). In fact soluble Aβ_1-42_ itself induces ATP release, auto-stimulating P2Y_4_ receptors in microglia, thus mediating its own clearance. Degradation of extracellular amyloid peptides is also performed by metallopeptidases such as matrix metalloproteinase 9 (MMP-9), whose secretion is upregulated following inhibition of the tonically active P2Y_14_ receptors (Kinoshita et al., 2013). These reports suggest that the loss of P2Y_2_ and P2Y_4_ receptors or an overactivation of P2Y_1_ and P2Y_14_ receptors alter the steady state levels of amyloid peptides leading to AD.

Both P2Y_2_ and P2Y_4_ receptors are preferentially expressed in perivascular astrocytes, and in response to exogenous ATP, mediate increased levels of cytosolic calcium within their end-feet processes (da Silva et al., 2009). As a result, P2Y receptors influence the permeability of blood-brain barrier through induction of endothelial nitric oxide synthase (eNOS). Activated glial cells also induce the expression of chemokines such as monocyte chemotactic protein 1 (MCP1) leading to the CNS recruitment of monocytes (Kim et al., 2011). Interestingly, MCP1 deficiency decreases microglial phagocytosis of Aβ oligomers, thus contributing to progressive amyloidosis (Kiyota et al., 2013).

UDP is the ligand of choice for P2Y_6_ receptors. Activated P2Y_6_ receptors trigger a change in microglial phenotype—from active motile/surveillance cells to active neuron devouring/phagocytic cells (Koizumi et al., 2007). The neurophagic activity of microglial cells is potentiated by TNF-α, LPS or Aβ peptides, and delayed through P2Y_6_ receptor antagonists (Neher et al., 2014). Furthermore, activated P2Y_6_ receptors block dilation of P2X_4_ receptor-mediated channels, shifting microglial phenotype from inflammatory to phagocytic cells (Bernier et al., 2013). Blocking of P2Y_6_ receptors increases neuronal survival suggesting that phagocytosis is not limited to degenerating neurons alone but non-specifically targets “stressed-but-otherwise-viable” neurons as well (Emmrich et al., 2013). Irrespective of the type of insult, the release of UDP signals microglia to initiate indiscriminate phagocytosis found in neurodegenerative disorders.

The migration of microglial cells to the site of injury is mediated by P2Y_12_ receptors (Haynes et al., 2006). In mice lacking P2Y_12_ receptors, microglia fail to polarize and migrate towards the lesion site while overactivation of P2Y_12_ receptors enhances neuroinflammation. As a result P2Y_12_^+/−^ mice show lesser severity of neuronal injury following cerebral ischemia compared to P2Y_12_^+/+^ littermates (Webster et al., 2013). Interestingly, loss of the transcriptional factor interferon regulatory factor 8 (IRF8) suppresses microglial chemotaxis (Masuda et al., 2014). Irf8^−/−^ microglia show reduced expression of P2Y_12_, P2X_4_ and adenosine A_3_ receptors—all involved in microglial activation and migration to the site of injury. As a result, Irf8^−/−^ mice are resistant to experimental autoimmune encephalitis (EAE)—a mouse model of multiple sclerosis (Yoshida et al., 2014).

ADP, formed by the activity of ectonucleotidases on extracellular ATP, is the preferred ligand for P2Y_13_ receptors. Ubiquitination at its C-terminal end leads to proteasomal degradation and poor surface expression (Pons et al., 2014). However its surface expression increases in response to oxidative stress and genotoxins such as cisplatin or UV irradiation (Morente et al., 2014). In the red blood cells, ADP-activated P2Y_13_ receptors show a negative feedback loop by inhibiting ATP release (Wang et al., 2005). Such a mechanism ensures additional regulation of extracellular ATP during neuronal injuries by restricting the lesion area such that undamaged and far away neurons which are exposed to ADP are not activated.

### Adenosine receptors

Enhanced neuroinflammation and microglial activity is a feature of A_1_ adenosine receptor knockout mice (A_1_AR^−/−^), suggesting that activation of A_1_ARs is neuroprotective under pathological conditions (Luongo et al., 2014). On the other hand, A_2A_ receptors facilitate glutamate release, and their association with ectonucleotidase, CD73, implies A_2A_ receptors are activated under pathological conditions when excess extracellular ATP is found (Augusto et al., 2013). Hence A_2A_ receptor antagonists such as caffeine limit the pathology of neurodegenerative disorders such as AD and PD. Interestingly, in mouse models of senescence, A_1_ARs significantly decrease with age while A_2A_ receptors increase with age (Castillo et al., 2009). A higher density of A_2A_ receptors in the putamen of PD patients also correlates with increasing motor symptoms (Varani et al., 2010). A_2A_ receptors induce microglial COX-2 expression (Fiebich et al., 1996) and inhibit astrocyte glutamate uptake by interacting with the α2 subunits of Na^+^/K^+^-ATPase (Matos et al., 2013). Though activation of A_1_ARs and inhibition of A_2A_ receptors provide neuroprotection in the adult brain, the opposite is true for the embryonic brain. Chronic hypoxia mediates accelerated maturation of oligodendrocyte progenitor cells leading to hypomyelination and ventriculomegaly in mice (Akundi and Rivkees, 2009). Deletion of A_1_ARs or early intervention with caffeine rescues embryos against hypoxia-mediated white matter injury (Back et al., 2006). Similarly, hypoxic ischemia-mediated brain damage was more profound in newborn A_2A_^−/−^ mice compared with their wildtype littermates (Adén et al., 2003). Similarly, it has been observed that A_2A_ receptor agonists show synergy with agonists of certain toll-like receptors, such as TLR2, 4, 7 and 9, in selectively upregulating the expression of vascular endothelial growth factor and downregulating the release of TNF-α (Pinhal-Enfield et al., 2003). Such a synergistic mechanism provides an angiogenic role for macrophages making it relevant in the aftermath of cerebral ischemia.

Adenosine A_3_ receptors mediate microglial process extension (Ohsawa et al., 2012). Agonists of A_3_ receptors thereby provide neuroprotection against ischemia (Choi et al., 2011). However, during chronic neuroinflammation microglia undergo process retraction through upregulation of adenosine A_2A_ receptors (Orr et al., 2009). A_2A_ receptor-dependent process retraction is also seen in the substantia nigra of mice treated with 1-methyl-4-phenyl-1,2,3,6-tetrahydropyridine (MPTP) for 5 days (Gyoneva et al., 2014). A_2A_ receptor-mediated loss of process extension (due to A_3_ receptors) and chemotaxis (due to P2Y_12_ receptors) thereby dampens microglial response to injury. Microglial cells also express α7 nicotinic acetylcholine receptor. Activation of these receptors attenuates neuroinflammation in a mouse model of MPTP (Liu et al., 2012). Coincidentally, nicotinic acetylcholine receptor stimulation mediates dopamine release in the rat striatum which is negatively regulated by agonists of adenosine A_2A_ receptors (Garção et al., 2013). These observations suggest that adenosine A_2A_ receptors regulate various neurotransmitter systems, and use of specific antagonists of A_2A_ receptors, therefore, show potential as therapeutic alternatives for PD (Threlfell et al., 2012).

## Targeting P2 receptors may be an attractive therapeutic approach over COX2 inhibition

Neuroinflammation in AD was promisingly approached through the use of NSAIDs (Szekely and Zandi, 2010). Following failures in clinical trials, the AD Anti-inflammatory Prevention Trial (ADAPT) group found that neither celecoxib nor naproxen prevented AD in adults with a family history of dementia (Breitner et al., 2013). The drawback of using COX inhibitors lies in the ubiquitous presence and role of its product, PGE_2_. PGE_2_ plays an important role in gastro-intestinal (GI secretion, bowel motility), cardiovascular (regulates blood pressure), renal (hemodynamics in the glomeruli), and reproductive (embryo implantation, uterine contraction) systems; and within the CNS, regulates body temperature, sleep-wake cycle, memory consolidation and synaptic plasticity. The relative contribution of either COX isoforms in mediating the above functions is unclear. Selective COX-2 antagonists were designed to target the excess PGE_2_ formed during neuroinflammatory episodes with the consideration that the more ubiquitous COX-1 would suffice for the production of physiological levels of PGE_2_. However, despite better gastrointestinal safety ratio, selective COX-2 inhibitors showed increased risks of myocardial infarction, stroke, systemic and pulmonary hypertension, and sudden cardiac death (Jüni et al., 2004). The multiple deaths led to the eventual withdrawal of COX-2 inhibitors such as rofecoxib leading to sweeping lawsuits and wider criticism of the drug licensing procedures.

In this scenario targeting P2 receptors, which modulate PGE_2_ synthesis, comes as a promising therapeutic possibility (Figure 1). The rat COX-2 promoter carries consensus sequences for transcription factors such as nuclear factor κB (NF-κB), NF-IL6, AP-1 and cAMP-responsive element (Tanabe and Tohnai, 2002). In addition, COX-2 can also be epigenetically regulated with hypermethylation responsible for its silencing in various types of cancer (Lodygin et al., 2005; Castells et al., 2006). Epigenetic contribution in the development of multiple sclerosis and neurodegenerative disorders is slowly being recognized, although evidences for such changes on P2 receptor genes is not yet known (Noh et al., 2012; Koch et al., 2013; Qureshi and Mehler, 2013). Furthermore, COX-2 is posttranscriptionally regulated as well. The human COX-2 mRNA contains at least 23 AU-rich elements (AREs) in the 3′-untranslated region (UTR) conferring to its instability (Shaw and Kamen, 1986). The interactions of ARE-binding protein with the 5′-methylguanosine cap-binding protein and polyadenosine tail-binding protein can either further stabilize the mRNA or lead to its degradation through recruitment of deadenylases (Dean et al., 2004). The p38 mitogen-activated protein kinase plays a critical role in the post-transcriptional regulation of several proinflammatory genes through controlling the phosphorylation status of these binding proteins (Clark et al., 2003). Other proteins that bind to COX-2 ARE and lead to its mRNA stabilization include the heat shock protein hsp70 (Kishor et al., 2013), and the RNA-binding protein HuR which inhibits the destabilization of COX-2 mRNA mediated by microRNA miR-16 (Young et al., 2012). Another regulator is the heterogeneous nuclear riboprotein A1 (hnRNP-A1) whose declining levels have been correlated with the severity of symptoms in various neurodegenerative diseases including AD (Bekenstein and Soreq, 2013). It was recently reported to regulate IL-6 transcription, with overexpression of hnRNP-A1 increasing IL-6 expression and knockdown leading to reduced IL-6 synthesis (Zheng et al., 2013). Post-transcriptional and post-translation regulation of various P2 receptors is not yet known, although the various alternate splicing mechanisms as shown in P2X7 (Adinolfi et al., 2010) and adenosine A1 receptor (Ren and Stiles, 1994) suggest their involvement. Transcription factors downstream of P2 receptor activation bind to most of the COX-2 promoter consensus sequences (Brautigam et al., 2005; Ferrari et al., 2006; Lenertz et al., 2011). P2X_7_ receptor antagonists present themselves as appropriate therapeutic alternatives to specific COX-2 inhibitors based on several evidences implicating them in neuroinflammation. Currently a few P2 receptor antagonists have advanced to clinical trials (Arulkumaran et al., 2011; North and Jarvis, 2013). It sets the stage for the potential role of P2 receptor-based anti-inflammatory drugs (PBAIDs) in targeting neuroinflammation.

A considerable roadblock in the design of PBAIDs is inherent in the diversity of P2 receptors. The conflicting reports on the neuroprotective and pro-inflammatory roles of various P2 receptors stems from the limited understanding of the actual types of receptors expressed, which may differ between cell types, species, age, and physiological status (Crain et al., 2009; Serrano et al., 2012). Nerve injury indiscriminately releases nucleotides of all kinds, including functionally opposing ones. The predominant ligand concentration depends on the distribution and activity of ectonucleotidases. Most studies utilize pharmacological agents which exhibit receptor promiscuity and have been characterized on purified homomers. However, not only surface P2 receptor density changes under pathological conditions, there is also the possibility of formation of heteromultimers (such as P2X_2/6_, or P2X_4/6_, and even P2X_2/4/6_) with altered ligand affinity and functions (Robinson and Murrell-Lagnado, 2013). The long and short splice variants of P2X_7_ receptor have opposing roles on cell growth and death (Adinolfi et al., 2010). Finally, an ideal PBAID has to overcome potential possibilities of receptor compensation. This was particularly evident in the failure of neuroprotection in P2X_7_^−/−^ mice where higher numbers of functionally compensating P2X_4_ receptors were found instead (Hracskó et al., 2011). In another example, P2Y_12_^−/−^ mice show delayed microglial response to injury; however, other signaling mechanisms did ensure that microglia reached the site of injury despite the delay (Haynes et al., 2006). The P2X_2_-P2X_5_ heterotrimer is functionally analogous to P2X_7_ receptors, including pore dilation, membrane blebbing and phosphatidylserine exposure (Compan et al., 2012). Such rich receptor diversity allows P2X receptors to functionally compensate the loss of other family members. Therefore, more studies are required to correctly identify the aggravating P2 receptor contributing to neuroinflammation. Because of the functional diversity of P2 receptors, PBAIDs should be carefully chosen to target the disease at the appropriate stage where benefit outweighs risk.

## Summary

**Figure 2:**
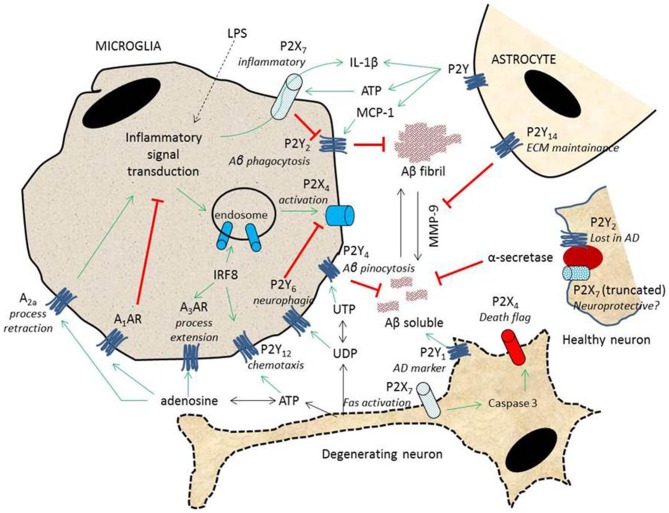
**P2 receptors modulate neuroinflammation**. A simplified model based on the literature mentioned in this review summarizes the interactions between neurons and glial cells. Pro-inflammatory signals modulate P2X_7_-mediated release of IL-1β and surface expression of P2X_4_ receptors in the presence of ATP released by degenerating neurons and reactive astrocytes. On the surface of neurons, P2X_7_ receptors mediate apoptosis with caspase 3-dependent expression of P2X_4_ receptors as “flags” for microglial engulfment. Microglial migration to sites of insult is mediated by P2Y_12_ and adenosine A_3_ receptors and its neurophagic activity through P2Y_6_ receptors. While A_1_ adenosine receptors inhibit general inflammatory pathways, A_2a_ receptors activate COX-2 as well as retract microglial processes. In healthy neurons, truncated P2X_7_ and P2Y_2_ receptors enhance α-secretase activity, preventing the formation of amyloid deposits. Amyloid formation is also immediately cleared through microglial phagocytosis, mediated by P2Y_2_ receptors; pinocytosis, through P2Y_4_ receptors; and activity of MMP-9, inhibited by the tonic activity of P2Y_14_ receptors.

Conventionally COX-2 has been a target in various inflammatory disorders. However, the failure of NSAIDs and selective enzyme inhibitors reveal the importance of COX-2 not only in various physiological activities but also in tissue repair following neuronal injury. The COX enzymes maintain a delicate balance of tissue scavenging and tissue repair during neuroinflammation. An imbalance could lead to excessive PGE_2_ activity leading to increased tissue damage or chronic inflammation. All cells within the vertebrate system upon damage (*hit one*) release large amounts of ATP (*hit two*) into the extracellular space. The effect of released ATP depends on the nature of two downstream factors—(1) the type of receptors present on cells within the vicinity of the injury; and (2) the distribution and activity of hydrolyzing ectonucleotidases. In large quantities, ATP potentiates the inflammatory reaction while other nucleotides have various modulatory roles in shaping the outcome of inflammation (summarized in Figure 2). PBAIDs aim to reduce the effect of *second hit* by targeting P2 receptors responsible for inflammation-enhancement rather than the COX enzymes mediating PGE_2_ synthesis. By not interfering with the COX system PBAIDs, unlike NSAIDs, retain the housekeeping functions of PGE_2_, but vastly reduce the pathology through P2 receptor inhibition. Identifying the target P2 receptor, and designing a selective PBAID, remains a challenge for future therapeutic successes in neuroinflammation. Surface expression of P2 receptors under certain pathological conditions may depend on epigenetic stimuli. Silenced P2 receptors which were once active during neural development could be reprogrammed in the event of tissue injury. A global study of P2 receptor density and mutations that affect their binding to specific nucleotides, may identify newer insights into the susceptibility of neurodegenerative disorders to specific populations. Furthermore, it is essential to understand the activity of various ectonucleotidases since the steady-state levels of various nucleotides have contrasting outcome in neuroinflammation. Therapeutically increasing the activity of specific ectonucleotidases following excessive ATP release is another approach to counter neuroinflammation. Finally, the two-hit hypothesis can also be extended to various other inflammatory disorders such as arthritis, toxin exposures including nerve gas poisoning, in the inflammatory model of cancer, and in psychological stress and depression. More studies in these areas will provide new roles for PBAIDs as effective anti-inflammatory drugs.

## Conflict of interest statement

The authors declare that the research was conducted in the absence of any commercial or financial relationships that could be construed as a potential conflict of interest.

## Abbreviations

AD: Alzheimer’s disease
ALS: amyotrophic lateral sclerosis
ATP: adenosine triphosphate
COX: cyclooxygenase
IL-1β: interleukin-1β
IRF8: interferon regulatory factor 8
LPS: lipopolysaccharide
MCP1: monocyte chemotactic protein 1
MMP-9: matrix metalloproteinase 9
NSAIDs: non-steroidal anti-inflammatory drugs
PARP: poly (ADP-ribose) polymerase
PBAIDs: P2 receptor-based anti-inflammatory drugs
PGE_2_: prostaglandin E_2_
PD: Parkinson’s disease
TNF-α: tumour necrosis factor α
UTP: uridine triphosphate
